# PREVALENCE OF LONELINESS AND ISOLATION AMONG PEOPLE WITH DEMENTIA AND THEIR CARERS

**DOI:** 10.1093/geroni/igz038.156

**Published:** 2019-11-08

**Authors:** Isla Rippon, Catherine Quinn, Anthony Martyr, Christina Victor, Fiona Mathews, Linda Clare

**Affiliations:** 1 Brunel University London, Uxbridge, Middlesex, United Kingdom; 2 University of Bradford, Bradford, England, United Kingdom; 3 University of Exeter, Exeter, England, United Kingdom; 4 Newcastle University, Newcastle, England, United Kingdom

## Abstract

People with dementia and carers may be vulnerable to loneliness and isolation. The IDEAL study includes two loneliness measures: 6 item de Jong Gierveld (DJG) scale (range 0-6) and a single-item self-report measure and the six-item Lubben social network scale (range 0-30). Full data are available for 1533 people with dementia for self-rated loneliness and for 1455 for the DJG scale and 1232 and 1195 carers respectively. For isolation complete data are available for 1489 people with dementia and 1252 carers. The prevalence of severe loneliness for people with dementia were 10% (self-rated) and 5% (DJG score 5+), approximately the population norm, and 15% and 18% respectively for carers. Most people with dementia or carers did not rate themselves as lonely ((79% and 71%) compared with 65% and 39% using the DJG scale. One third, 35%, of people with dementia were at risk of isolation compared with 18% of carers.

